# Editorial: Immunogenomics of Solid Organ and Hematopoietic Stem Cell Transplantation

**DOI:** 10.3389/fimmu.2022.878314

**Published:** 2022-03-16

**Authors:** Yongxia Wu, Julien Zuber, Jianing Fu

**Affiliations:** ^1^ Department of Microbiology and Immunology, Medical College of Wisconsin, Milwaukee, WI, United States; ^2^ Department of Kidney Transplantation, Necker Hospital and Human Lymphohematopoiesis lab, INSERM UMR 1163, IMAGINE Institute, Paris University, Paris, France; ^3^ Columbia Center for Translational Immunology, Department of Medicine, Columbia University, New York, NY, United States

**Keywords:** immunogenomics, SOT, HSCT, next-generation sequencing, GVHD, graft rejection, immune tolerance

Alloreactivity is caused by the extensive difference in polymorphic genes between allogeneic donor and recipient, primarily in the major histocompatibility complex (MHC). As key mediators of alloresponses, alloreactive T cells are educated by self MHC and thus acquire the ability to recognize non-self MHC, leading to graft rejection and graft-versus-host-disease (GVHD), in solid organ transplantation (SOT) and hematopoietic stem cell transplantation (HSCT), respectively (1). Through production of donor-specific antibodies, B cells also perpetuate alloresponses to cause tissue injury after SOT (2) and contribute to pathogenesis of chronic GVHD following allogeneic HSCT (3). Both T and B cells express an enormous diversity of antigen-specific receptors and form the basis of the host’s ability to respond to infectious threats. Recent advances in next-generation sequencing technologies (4) have enabled new opportunities to provide a precise picture of the tremendously diverse immune repertoire and to tackle fundamental questions of alloimmunity (5). In this Research Topic, we bring together four novel Original Research articles (Tinel et al.; Aschauer et al.; Wu et al.; Okamura et al.) and three comprehensive Review articles (Shi et al.; Tian et al.; Fu et al.) on immunogenomics of SOT and HSCT that include both animal and human studies.

Shi et al. provides an insightful review of genomic-based approaches to the study of transplant rejection, including microarray, RNA sequencing (RNA-seq), and spatial transcriptomic techniques. This review traces the developmental history of these approaches and extends it to the current fast-paced field of emerging technologies, which includes the integration of single cell RNA-seq with T cell receptor (TCR) and B cell receptor (BCR) sequencing to profile immune repertoires, with mass cytometry and featured barcode antibodies to measure protein expression, and with chromatin sequencing to explore gene regulatory networks. The authors discuss the advantages and limitations of each approach. Application of these analysis tools will contribute to a fundamental understanding of the alloresponse and likely promote novel therapeutic options to overcome rejection and GVHD.

To investigate the role of microRNAs (miRNAs) in antibody-mediated rejection (AMR) after SOT, Tinel et al. performed miRNA and mRNA profiling of kidney allograft biopsies to reveal new pathways involved in microvascular Inflammation (MVI), the main histological injury associated with AMR. This study identifies six differentially-expressed miRNAs that are correlated with the intensity of MVI. mRNAs/miRNAs interplay analysis further elucidates the crosstalk between renal-resident and allograft-infiltrating cell subsets and suggests that epithelial, rather than endothelial, metabolism modifications occur during AMR. This study illustrates the great potential of multi-omics to decipher rejection mechanisms.

TCR repertoire diversity and turnover dynamics are related to rejection and tolerance in SOT (6). The presence of donor-reactive T cells, defined by mixed lymphocyte reaction (MLR) combined with high throughput TCR-seq, contributes to T-cell-mediated rejection (TCMR). Tian et al. reviews recent advances in TCR-seq and computational tools and discusses the potential of using TCR-seq to profile alloreactive T cell repertoires. Defining and tracing donor- and recipient-reactive TCRs may reveal the fingerprint of alloreactive T cells, providing valuable indication of graft rejection, acceptance and treatment response after SOT.

Frequencies of circulating donor-reactive T cells were elevated in kidney transplant patients receiving conventional immunosuppression (5, 7). However, it is unclear whether circulating donor-reactive T cell clones indeed reflect graft-infiltrating TCRs in rejection episodes. Aschauer et al. analyzes donor-reactive TCRs in pre-transplant blood and post-transplant kidney biopsies in the presence and absence of TCMR to find little repertoire overlap in these two sites. While circulating donor-reactive repertoire is increased in both groups, donor-reactive T cells in kidney are only enriched during a TCMR episode with substantially diverse TCRs, indicating the capability to respond against a variety of epitopes in the allograft.

Fu et al. further discusses the use of TCR-seq to discern the factors behind human T cell repertoire development and how this approach can be used in combination with human immune system mouse models to understand human repertoire selection. The article explains current understanding of the propensity of alloreactive TCRs as a consequence of thymic selection. It also notes the limitations of techniques historically used to study human TCR repertoires and provides descriptions of innovative tools the authors are utilizing (6–12) to unravel donor-host interactions from the perspective of alloreactive T cells. New insights into human allograft rejection and tolerance obtained with the MLR and high throughput TCR-seq method in combination with single cell transcriptional analyses are discussed (13, 14).

T cells are considered as not only the main culprit behind TCMR after SOT, but also the driving force of GVHD after allogeneic HSCT (15). Recovery of TCR diversity in patients after HSCT is impacted by the source of donor grafts, tumor relapse, GVHD development, and steroid response (16–18). By extensively examining T cell repertoire features in lymphoid and parenchyma organs after allogeneic and syngeneic HSCT in mice, Wu et al. demonstrates: 1) TCR diversity is narrowed in allogeneic T cells; 2) top dominant T cell clones are highly shared across circulation and GVHD target organs in allogeneic recipients; 3) clonal expansion of rare rearrangements from pre-transplant donor T cells may account for the sharing of a few clones among allogeneic recipients. Their findings illustrate immune repertoire sequencing-based methods as a novel personalized strategy to guide diagnosis and therapy in GVHD.

Along with graft rejection and GVHD, transplant-associated thrombotic microangiopathy (TA-TMA) is also a life-threatening medical condition. Although pathogenic variants in complement regulatory genes have been reported as genetic susceptibility to TA-TMA in only a minority of patients (19, 20), growing evidence suggests complement activation in this condition, irrespective of genetic predisposition (21). Okamura et al. reports that high levels of complement factor Ba a week after HSCT predict the occurrence of TA-TMA and related non-relapse mortality. This finding, once confirmed in a broader cohort, will lay the groundwork for highly-tailored and complement-targeted therapeutics. C5 blockade—which already revolutionized outcomes of atypical hemolytic uremic syndrome (22), another form of TMA—holds promise (23).

This Research Topic of articles provides an in-depth review of current understanding of alloresponses after transplantation in preclinical and clinical settings from the immunogenomics perspective and encourages future investigations in this field.

## Author Contributions

YW, JZ, and JF collected articles and wrote the editorial review. All authors contributed to the review and approved the submitted version.

## Funding

YW was supported by R01 CA258440 from NIH/NCI (PI: Xue-Zhong Yu). JZ was supported by the Fondation Emmanuel Boussard, IMAGINE Institute, DIM-Thérapie Génique/Région Ile-de-France, Agence de la Biomédecine, and Agence Nationale de la Recherche (DAISY, ANR-20-CE18-0004). JF was supported by a Congressionally Directed Medical Research Program (CDMRP) Discovery Award W81XWH-20-1-0159 funded by the Department of Defense (DoD), a R21 grant AI166069 supported by NIH/NIAID and Nelson Faculty Development Awards UR011630-01 and UR011630-02 from the Nelson Family Transplantation Innovation Award Program at Columbia University Irving Medical Center.

## Conflict of Interest

The authors declare that the research was conducted in the absence of any commercial or financial relationships that could be construed as a potential conflict of interest.

## Publisher’s Note

All claims expressed in this article are solely those of the authors and do not necessarily represent those of their affiliated organizations, or those of the publisher, the editors and the reviewers. Any product that may be evaluated in this article, or claim that may be made by its manufacturer, is not guaranteed or endorsed by the publisher.
